# mTOR Complex 1 Implicated in Aphid/*Buchnera* Host/Symbiont Integration

**DOI:** 10.1534/g3.118.200398

**Published:** 2018-07-26

**Authors:** Edward B. James, Honglin Feng, Alex C. C. Wilson

**Affiliations:** Department of Biology, University of Miami, Coral Gables, FL 33146

**Keywords:** mTORC1, symbiosis, *Myzus persicae*, transcriptome

## Abstract

Obligate nutritional endosymbioses are arguably the most intimate of all interspecific associations. While many insect nutritional endosymbioses are well studied, a full picture of how two disparate organisms, a bacterial endosymbiont and a eukaryotic host, are integrated is still lacking. The mTOR pathway is known to integrate nutritional conditions with cell growth and survival in eukaryotes. Characterization and localization of amino acid transporters in aphids suggest the mTOR pathway as a point of integration between an aphid host and its amino acid-provisioning endosymbiont *Buchnera aphidicola*. The mTOR pathway is unannotated in aphids and unstudied in any nutritional endosymbiosis. We annotated mTOR pathway genes in two aphid species, *Acyrthosiphon pisum* and *Myzus persicae*, using both BLASTp searches and Hidden Markov Models. Using previously collected RNAseq data we constructed new reference transcriptomes for bacteriocyte, gut, and whole insect tissue for three lines of *M. persicae*. Annotation of the mTOR pathway identified homologs of all known invertebrate mTOR genes in both aphid species with some duplications. Differential expression analysis showed that genes specific to the amino acid-sensitive mTOR Complex 1 were more highly expressed in bacteriocytes than genes specific to the amino acid-insensitive mTOR Complex 2. Almost all mTOR genes involved in sensing amino acids showed higher expression in bacteriocytes than in whole insect tissue. When compared to gut, the putative glutamine/arginine sensing transporter ACYPI000333, an ortholog of SLC38A9, showed 6.5 times higher expression in bacteriocytes. Our results suggest that the mTOR pathway may be functionally important in mediating integration of *Buchnera* into aphid growth and reproduction.

Two species living together in symbiosis can reciprocally impact each other’s evolution (Ehrlich and Raven 1964). The most intimate symbioses are those where one species resides inside the cells of the second species in obligate endosymbiosis (Wernegreen 2004). In plant-sap feeding insects such obligate endosymbioses are typically nutritional and feature hosts that require their endosymbiont for reproduction, and endosymbionts that are unable to survive outside their host. Despite the apparent harmony between members of these nutritional endosymbioses the partners must balance conflict and cooperation for the relationship to persist (Bennett and Moran 2015; Wernegreen 2004). The host must not completely reject the intracellular symbiont population but instead must provision the metabolites required for endosymbiont survival. In turn, the endosymbiont must persist within the host cell without imposing a fatal cost upon the host. Members of an endosymbiosis face unique and separate selection pressures that govern their evolution as discrete species, while existing in a robust state of integration (Bennett and Moran 2015; Keeling and McCutcheon 2017). Despite the evolutionary, ecological, agricultural and economic importance of many insect obligate nutritional endosymbioses a full picture of how these relationships are functionally regulated by their members is lacking. Therefore an understanding of how these endosymbiotic interactions evolved, are maintained, and indeed how they might be targeted for human control is also wanting.

The most-studied insect model of a host endosymbiont relationship is that of aphids and their endosymbiont, *Buchnera aphidicola. Buchnera* are housed within specialized aphid organs called bacteriomes, where they are contained inside bacteriocyte cells (Baumann *et al.* 1995). The two species are integrated to such an extent that *Buchnera* is unable to produce their own outer-membrane, and instead it is assumed to be produced by the aphid host (Shigenobu *et al.* 2000). Another feature of the integration includes the loss in both aphid hosts and *Buchnera* of amino acid biosynthesis genes. Such gene losses have sometimes involved the complete degradation of pathways like the TCA cycle in *Buchnera*, (rendering *Buchnera* incapable of producing glutamate and aspartate), and the urea cycle in aphids (rendering aphids incapable of producing arginine) (International Aphid Genomics Consortium 2010; Shigenobu *et al.* 2000). Other times gene losses have not eliminated a pathway but rather fragmented it in such a way that completion of the metabolic pathway requires the contribution of enzymes encoded by genes in both the host and the symbiont genome (Hansen and Moran 2011; Price *et al.* 2015; Wilson *et al.* 2010).

Multiple points of regulation of the collaborative biosynthesis of amino acids in aphids have recently been identified. First, *Buchnera*’s amino acid production has been proposed to be in part regulated by host-controlled supply of the precursor glutamine via a glutamine specific amino-acid transporter which is highly expressed in bacteriocytes and competitively inhibited by arginine, a *Buchnera* produced end-product amino acid (Price *et al.* 2014; Price *et al.* 2015). Second, microbial small RNAs that are conserved across *Buchnera* from different aphid species have been proposed to function in *Buchnera* gene regulation (Hansen and Degnan 2014). In particular, life-stage dependent differential expression of amino acid biosynthesis pathways within *Buchnera* has been hypothesized to result from post-transcriptional small RNA regulation within *Buchnera* (Hansen and Degnan 2014). Third, microRNAs (miRNAs) encoded in aphid genomes have been found to show bacteriocyte-specific expression (Feng *et al.* 2018). Remarkably, of the 14 miRNAs highly or differentially expressed within aphid bacteriocytes, ten have previously been characterized to play roles in host/microbe interactions (Feng *et al.* 2018). The emerging picture of host/endosymbiont regulation is one of multiple concurrent and overlapping mechanisms of integration. While some of these mechanisms have been characterized we do not yet have a full mechanistic understanding of host/endosymbiont integration. In particular, the mechanisms that link *Buchnera* amino acid output, the currency of this nutritional endosymbiotic relationship, to symbiont growth and proliferation are yet to be identified.

The highly conserved mechanistic target of rapamycin (mTOR) signaling pathway (Figure 1) links a cell’s intracellular amino acid availability, extracellular growth factors, and available energy levels to cellular responses that include protein synthesis, lipid synthesis, cell proliferation, cytoskeleton organization, autophagy, and mitochondrial regulation and proliferation (Crespo and Hall 2002; Laplante and Sabatini 2012; Morita *et al.* 2015; Sarbassov *et al.* 2004; Shimobayashi and Hall 2016; Goberdhan *et al.* 2016). This signaling pathway centers around two complexes: mTOR Complex 1 (mTORC1) and mTOR Complex 2 (mTORC2). The complexes form around the mTOR protein, but differ in protein composition such that each complex has distinct functions. mTORC1 is activated in response to sufficient levels of amino acids and responds by signaling for protein, nucleotide, and lipid synthesis, and blocking autophagy; mTORC1 allows cellular growth when nutritional conditions will support growth (Goberdhan *et al.* 2016). The genes and complexes upstream of mTORC1 and mTORC1 itself are referred to in this paper as the amino acid sensing pathway. In contrast, mTORC2 integrates growth factors and other extracellular signals with cell survival and cytoskeleton organization (Ebner *et al.* 2017; Wullschleger *et al.* 2006). The mTOR pathway represents a conspicuous candidate for integrating amino acid-provisioning, endosymbiont growth and population size to their metabolic output within host tissues. Currently, mTOR is unannotated and unstudied in any nutritional endosymbiosis.

**Figure 1:**
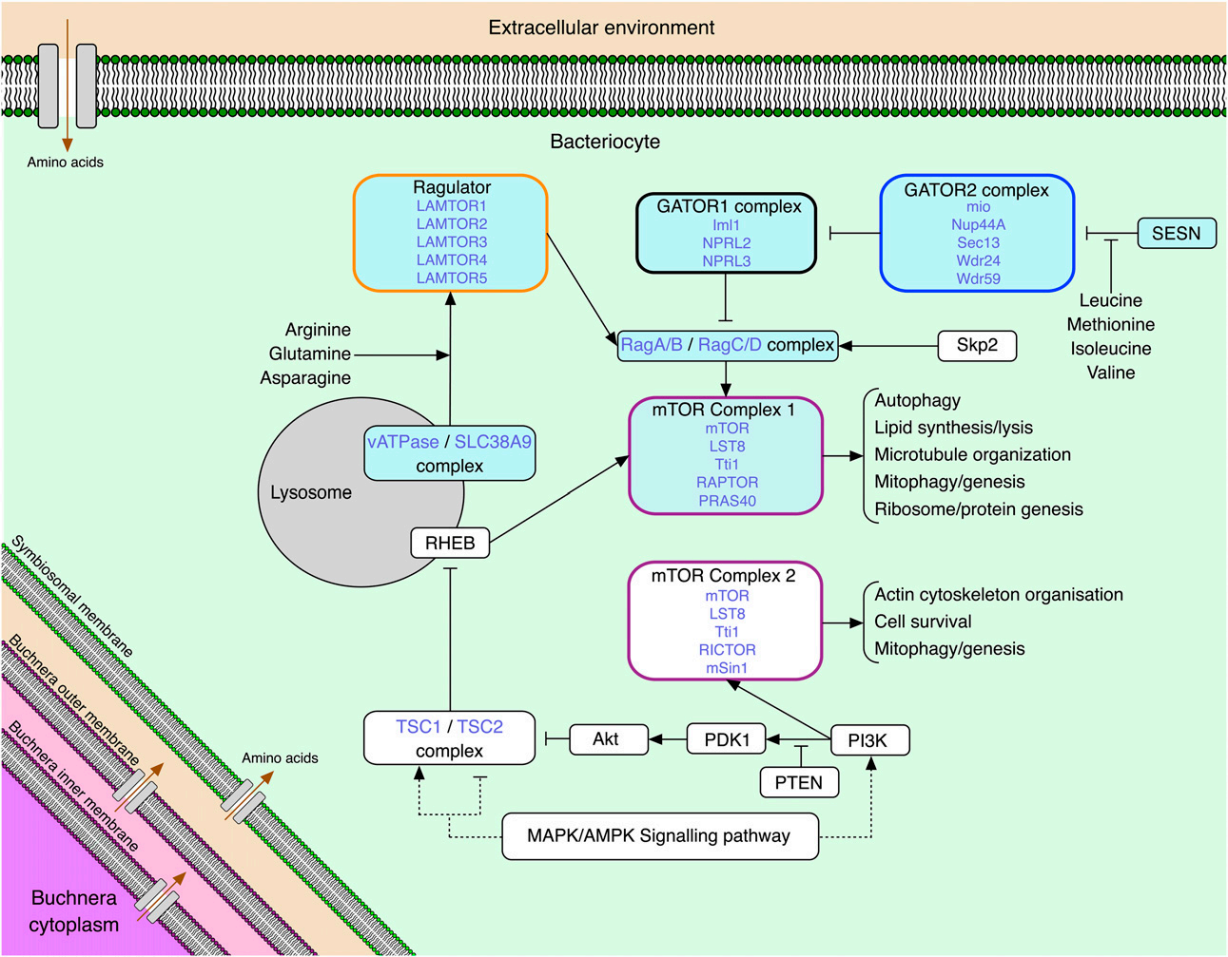
The mTOR pathway within an aphid bacteriocyte. The names of complexes are written in black at the top of their box, with their constituent genes in blue. Orthologs to known amino acid sensing genes and complexes are shaded blue. (Amino acid inputs: Rebsamen *et al.* 2015; Wolfson *et al.*, 2016)

In this study we present an annotation of the central components of the mTOR pathway and its associated amino acid-sensing cellular machinery in the aphids *Myzus persicae* and *Acyrthosiphon pisum*. In addition, we re-analyze previously collected RNAseq data generated from *M. persicae* bacteriome, gut, and whole insect tissue reporting differential and ranked expression of mTOR pathway components from three genetically distinct *M. persicae* lines.

## Materials and Methods

Annotation of the mTOR pathway in *Myzus persicae* and *Acyrthosiphon pisum* was done using both BLASTp searches in which annotated mTOR proteins from *Drosophila melanogaster* were used as queries (accessed from FlyBase), and Hidden Markov Models of mTOR-associated genes (accessed from PantherDB (Mi *et al*. 2016)) against the *M. persicae* G006 (NCBI taxid: 13164) and *A. pisum* LSR1 (NCBI taxid: 7029) reference genomes. Gene models and gene duplications identified using our annotation pipeline were validated using PhylomeDB to confirm orthology and paralogy relationships (Huerta-Cepas *et al.* 2014).

We used the RNAseq that was generated by Duncan *et al.* 2016 to construct reference transcriptomes from three different *M. persicae* genotypes based on *M. persicae* G006 v1.0 official gene set from aphidbase (bipaa.genouest.org/is/aphidbase). We used RNAseq generated in Feng *et al.* 2018 and Jaquiéry *et al.* 2018 to produce reference transcriptomes for *A. pisum* based on the 2.1b official gene set on aphidbase. We did not use the previous *de novo* transcriptomes because they were unsuitable for differentiating between known duplicated genes. For *M. persicae* bacteriocyte and gut tissue transcriptomes, 300 aphids were dissected and pooled and 10 aphids were used for whole insect transcriptomes as described in Duncan *et al.* 2016. Quality control was carried out in FastQC (Andrews 2010) along with the TrimGalore v0.4.3 (Kruger 2012) package. Reads were aligned using the Hisat2 v2.0.0 (Kim *et al.* 2015) package. Differential expression analysis was carried out using the nbinomtest() function in DESeq2 in SeqMonk (Andrews 2007; Love *et al.* 2014).

For additional more detailed methods please see supplemental material.

### Statistical Analysis

A principle component analysis was performed with ggplot2 in R (R Development Core Team 2010; Wickham 2009) on the *M. persicae* transcriptomes from all genotypes and tissues after quality control to ascertain if there were any batch effects. Transcriptomes grouped clearly by tissue type rather than genotype so batch effect removal was deemed unnecessary (Figure S1).

### Data availability

*M. persicae* RNAseq data can be accessed on the NCBI Sequence Read Archive under BioProject PRJNA296778. *A. pisum* RNAseq data (see discussion) can be accessed on the NCBI Sequence Read Archive under BioProject PRJNA315109 and PRJNA385573. Aphid lines are available upon request. Supplemental files available at FigShare. Figure S1 contains PCA analysis of transcriptomes. Figure S2 contains the complete comparison of mTOR genes between gut and bacteriocyte transcriptomes. Figure S3 contains the synteny-based alignment of duplicated genes between *A. pisum* and *M. persicae*. Table S1 contains the sequence identity of mTOR genes in *A. pisum* and *M. persicae*. Sequence data are available at aphidbase (https://bipaa.genouest.org/is/aphidbase/). Table S2 gives the sequences and HMMs used for gene annotation. These can be accessed at flybase (http://flybase.org/), and on pantherDB (http://pantherdb.org/) and eggnog mapper (Huerta-Cepas *et al.* 2016) (http://eggnogdb.embl.de).

File S1 contains additional more detailed methods used in this study. Supplemental material available at Figshare: https://doi.org/10.25387/g3.6855089.

## Results

### Aphids show novel duplications in the highly conserved mTOR pathway

**Figure 2:**
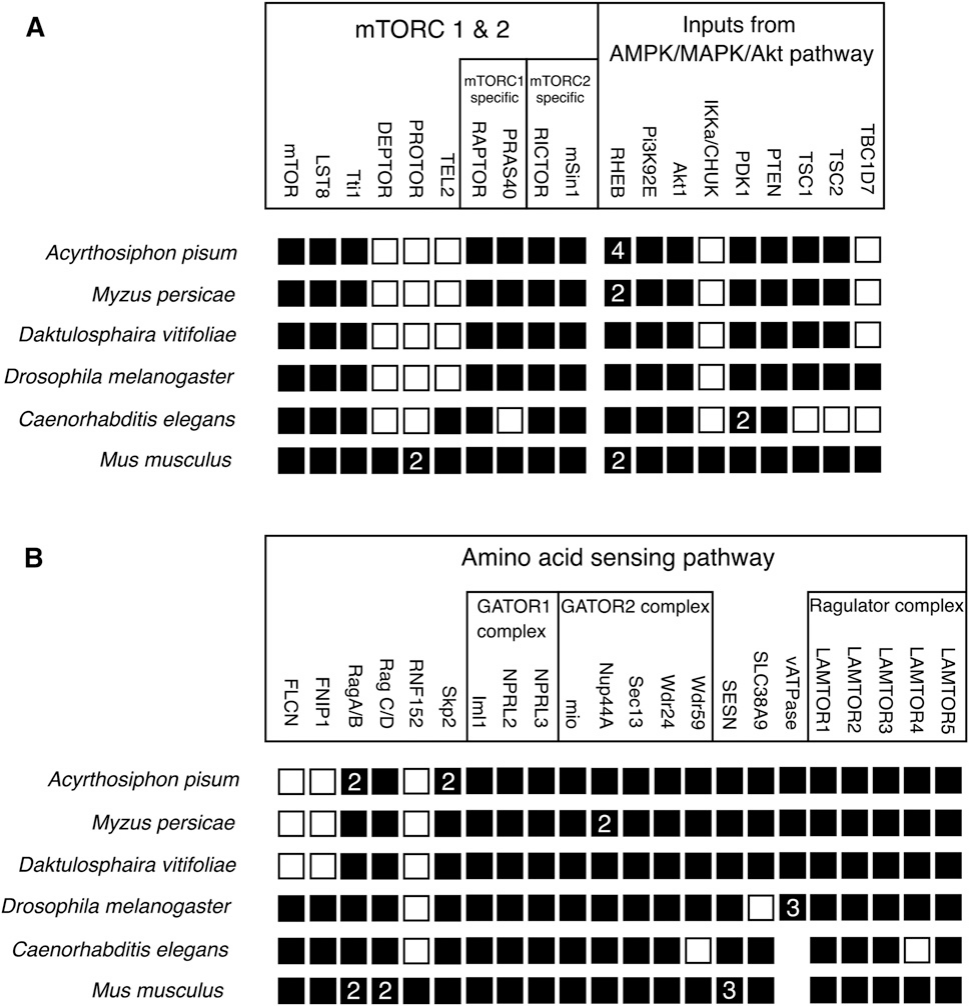
Presence and absence of genes in (A) the mTOR complexes, and inputs from the AMPK/MAPK/Akt pathway and (B) the mTOR amino acid sensing pathway. Genes were identified by Hidden Markov Models, and confirmed by PhylomeDB and KEGG. A filled square indicates the gene is present in the species’ genome. Where duplications have occurred the number of gene copies is shown in the square. An empty square indicates the gene is absent from a species’ genome. The empty spaces for vATPase indicates that genes could not be confidently annotated using Hidden Markov Models. The HMMs were selected to perform annotation in aphid species, and as the vATPase family contains many genes annotation using these HMMs was not reliable for *C. elegans* or *M. musculus*.

*A. pisum* and *M. persicae* both retain the mTOR genes that are widely conserved across invertebrates (Figure 2 A & B, Table S1). Genes missing from the aphid genomes (DEPTOR, PROTOR, Tel2, IKKα, TBC1D7, FLCN, FNIP1, and RNF152) are mostly found only in vertebrates. Both aphid species show gene duplications. We identified four RHEB orthologs, and two orthologs of both Rag A/B, and Skp2 in the *A. pisum* genome that are not shared with either the grape phylloxera, *Daktulosphaira vitifoliae* or the fruitfly, *Drosophila melanogaster*. We identified two RHEB orthologs, and two Nup44A orthologs in the *M. persicae* genome that are not shared with either *D. vitifoliae* or *D. melanogaster*. Based on their synteny, two of the RHEB duplications are inferred to have been present in the common ancestor of *A. pisum* and *M. persicae* (Figure S3A).

### The mTOR pathway is expressed in M. persicae bacteriome tissue

**Figure 3:**
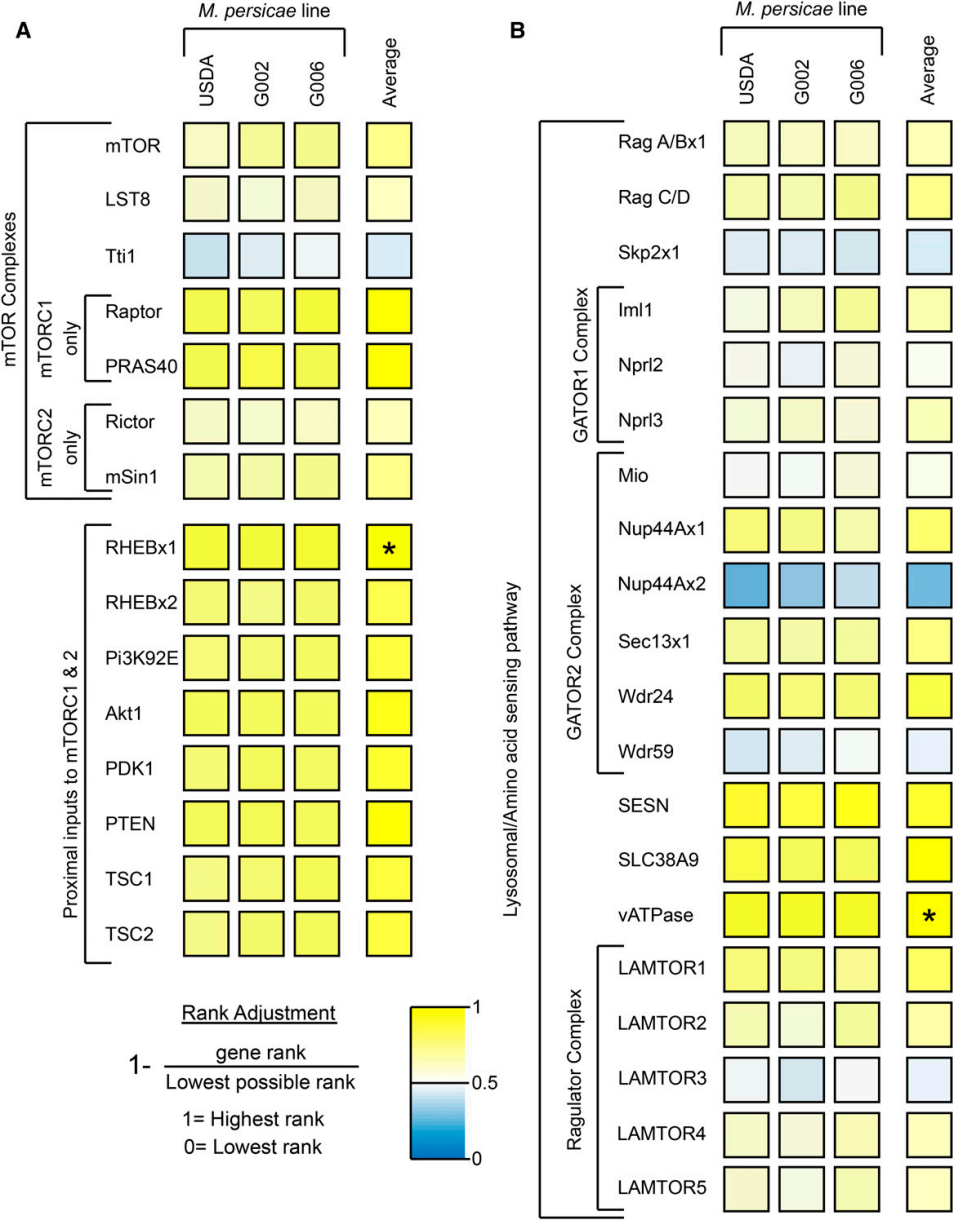
Ranked expression heatmap of (A) mTOR complexes, inputs from the AMPK pathway and (B) the mTOR amino acid sensing pathway in three genotypes of *M. persicae*. Ranks were adjusted to a scale of 1 – 0, with a score of 1 indicating the highest ranked genes, and a score of zero indicating the lowest ranked genes. Yellow indicates a rank in the top half of genes, while blue indicates a rank in the bottom half. Asterisks indicate the top 10% of expressed genes. Aphid duplications are identified with the suffix ‘x1’ or ‘x2’. Total number of genes ranked: USDA: 11720, G002: 11672, G006: 12030.

We identified all *M. persicae* mTOR-associated genes in the bacteriocyte transcriptomes of three genetically distinct *M. persicae* lineages (Figure 3). Most, 28 of 34, mTOR-associated genes ranked in the top half of expressed genes, with one of two RHEB orthologs and vATPase ranking in the top 10% of expressed genes. The mTORC1-specific genes: Raptor and PRAS40, are more highly expressed in bacteriocyte tissue than the mTORC2-specific genes, Rictor and mSin1 (Figure 3A).

### mTOR genes show bacteriocyte-specific expression patterns

**Figure 4:**
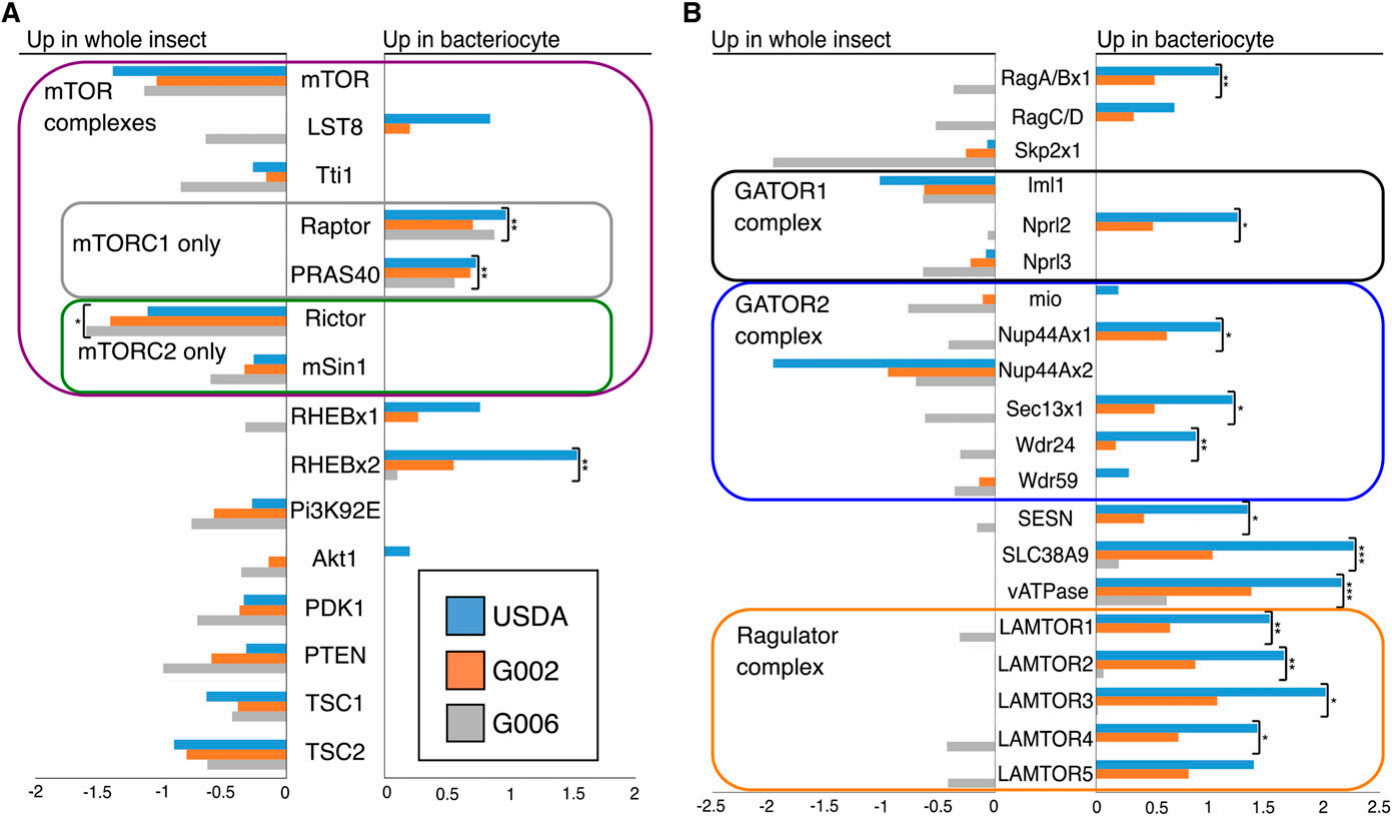
Log_2_ fold change in RPM between bacteriome and whole insect tissue for three genotypes of *M. persicae* in (A) mTOR complex genes, inputs from the AMPK pathway, and (B) mTOR amino acid sensing pathway. Positive numbers indicate higher expression in bacteriome, negative numbers indicate higher expression in whole insect. Asterisks indicate FDR adjusted significant results. * shows *P* < 0.05, ** shows *P* < 0.001, *** shows *P* < 0.00001

**Figure 5:**
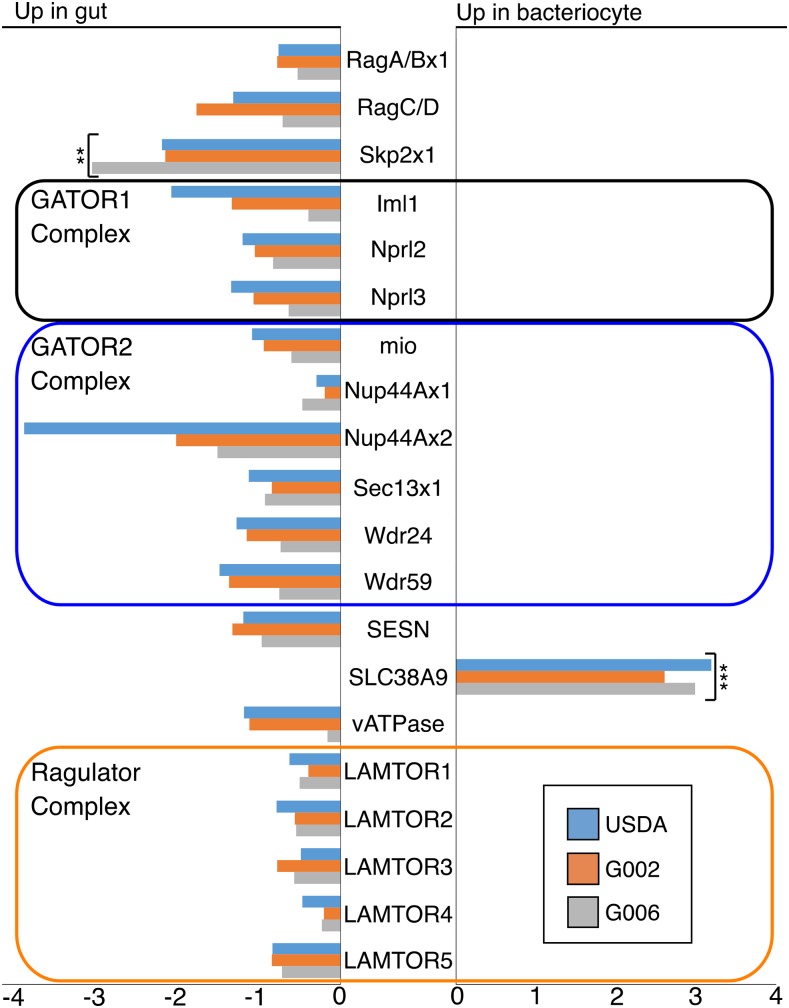
Log_2_ fold change in RPM between bacteriome and gut tissue of the mTOR amino acid sensing pathway in three genotypes of *M. persicae*. Positive numbers indicate higher expression in bacteriome, negative numbers indicate higher expression in gut. Asterisks indicate FDR adjusted significant results. * shows *P* < 0.05, ** shows *P* < 0.001, *** shows *P* < 0.00001

In *M. persicae* mTORC1 the genes, Raptor and PRAS40, are more highly expressed in bacteriome tissue than in whole insect tissue, while the mTORC2-specific genes, Rictor and mSin1, are less highly expressed in bacteriome tissue than in whole insect tissue (Figure 4A). The majority of genes (12 of 20) in the mTOR amino acid sensing pathway, including all members of the Ragulator complex (LAMTOR1-5), Sestrin (SESN), putative arginine transporter SLC38A9, and vATPase (Figure 4B) show significantly higher expression in bacteriome tissue than in whole insect tissue. Notably, the putative arginine transporter SLC38A9 is also more highly expressed in bacteriome tissue than in gut tissue (Figure 5).

## Discussion

### Aphids have a complete invertebrate mTOR pathway

Annotation of the mTOR pathway in aphids shows that, with a few exceptions, they possess the mTOR related genes that would be expected in invertebrates, and that the aphid complement of mTOR related genes is similar to that of two other arthropods, the grape phylloxera, *D. vitifoliae* (a close relative of aphids, but lacking an endosymbiont) and the fruitfly, *D. melanogaster* (Figure 2). Given that the mTOR pathway is heavily conserved in all eukaryotes (Hall 2008), it is unsurprising that aphids share many gene orthologs with the non-arthropods *Caenorhabditis elegans* and *Mus musculus*. Most genes absent from the aphid mTOR pathway are commonly absent from other annotated invertebrates (Figure 2), although TBC1D7, FLCN, and FNIP may represent more recent gene losses within Sternorrhyncha or Hemiptera (the insect suborder and order to which aphids belong) as these genes are present in the fruit fly, *D. melanogaster*. The loss of these three genes is not expected to interrupt the functions of the genes present, although the losses of FLCN and FNIP represent loss of an amino acid-sensitive mTORC1 associated complex (Tsun *et al.* 2013).

### Aphids show novel duplications within the mTOR pathway

The genomes of both, *M. persciae* and *A. pisum* showed duplications that are not present in other invertebrates including the closely related grape phylloxera, *D. vitifoliae*. *Acyrthosiphon pisum* contains four RHEB orthologs, two of which are shared with *M. persicae* and assumed to be ancestral (Figure S3). In addition, *A. pisum* has two Rag A/B, and Skp2 orthologs, and *M. persicae* two Nup44A orthologs. All of these duplicated genes with the exception of the two RHEB orthologs unique to *A. pisum* are detected in wingless parthenogenetic female aphid transcriptomes (Feng *et al.* 2018; NCBI BioProject PRJNA315109). The two RHEB orthologs unique to *A. pisum* were not detected in the transcriptomes associated with this study but are found in an alate male aphid transcriptome (data from Jaquiéry *et al.*, 2018. BioProject PRJNA385573), suggesting male-specific function associated with these *A. pisum*-specific duplications (ACYPI005487 and ACYPI006392).

Acquisition of novel genomic material into host genomes by processes that include gene duplication and lateral gene transfer appears to be a feature of coevolution in host/endosymbiont systems (Wilson & Duncan 2015). Previous work in obligate sap-feeding insects, including aphids has shown that duplications in the amino acid-auxin-permease family (AAAP) of transporters has occurred after endosymbiont colonization while work with the amino acid-polyamine-organocation (APC) transporter family has shown that duplications have occurred both before and after endosymbiont colonization (Duncan *et al.*, 2016). While duplications that occurred before endosymbiont colonization cannot have been driven by symbiosis, post colonization duplication events may have been driven by host/endosymbiont coevolution, and indeed many duplicated amino acid transporters from both the AAAP and APC transporter families show bacteriocyte-specific expression (Duncan *et al.*, 2016).

### Amino acid-sensitive mTORC1 appears to be more important in bacteriomes than the amino acid-insensitive mTORC2

The mTOR pathway can take multiple signals as inputs, with amino acid-sensing signals being parsed through mTORC1, and growth factor signals through mTORC2 (Goberdhan *et al.* 2016; Laplante and Sabatini 2012; Shimobayashi and Hall 2016). The higher expression of mTORC1-specific genes compared with mTORC2-specific genes both within bacteriocytes, and in comparison to whole insect tissue suggests that the role of mTORC1 in bacteriocyte tissue is more important than that of mTORC2 (Figures 3 & 4A). Bacteriocytes are specialized cells that function to house *Buchnera*, a bacteria with a genome streamlined for essential amino acid production (Moran and Mira 2001; Wernegreen 2002). Both *A. pisum* and *M. persicae* strains of *Buchnera* have undergone severe genome reduction, losing DNA repair mechanisms, cell membrane and surface synthesis pathways, and some non-essential amino acid synthesis pathways (Jiang *et al.* 2013; Shigenobu *et al.* 2000). Based on genomic analyses the main function retained by *Buchnera* for host benefit is production of host-essential amino acids and vitamins. Given the wide range of inputs the mTOR pathway is able to take, it is interesting that the amino acid-sensitive mTORC1 shows high expression within bacteriocytes. The high expression of mTORC1-specific genes suggests that amino acid sensing through mTORC1 plays a role in mediating the relationship between aphids and *Buchnera*. mTORC1 has two points of amino acid integration: Sestrin and SLC38A9. Sestrin activates mTORC1 via the GATOR complexes (Figure 1), and detects leucine, methionine, isoleucine and valine (Figure 1) (Saxton *et al.* 2016; Wolfson *et al.* 2016). SLC38A9, which only affects mTORC1 when it colocalizes with vATPase, activates mTORC1 when co-localized with vATPase via the Ragulator complex (Figure 1), and is responsive to glutamine, arginine, and asparagine (Rebsamen *et al.* 2015).

### Collaboratively synthesized amino acids could be integrated into mTORC1 signaling by a sensitive Sestrin gene

Sestrin, a stress-induced protein capable of inhibiting the GATOR2 complex (Figure 1; Wolfson *et al.* 2016), senses four amino acids: leucine, isoleucine, methionine and valine by binding to them and thereby interfering with Sestrin’s GATOR2 inhibition mechanism (Wolfson *et al.* 2016). In aphids these four amino acids are four of five amino acids that are hypothesized based on genomic analysis (Shigenobu *et al.* 2000; Wilson *et al.* 2010) and some experimental validation (Russell *et al.* 2013) to be products of aphid/*Buchnera* collaborative amino acid biosynthesis. Biosynthesis of three of these four amino acids, isoleucine, leucine and valine, appears to takes place almost entirely within *Buchnera*, with the exception of the final step in each of these pathways which is proposed to be carried out by branched-chain-amino-acid transaminase (BCAT), a eukaryotic gene encoded in the aphid genome, whose functional ortholog is notably absent in *Buchnera* (International Aphid Genomics Consortium 2010; Jiang *et al.* 2013; Price *et al.* 2015; Russell *et al.* 2013; Wilson *et al.* 2010). This evidence for a host-catalyzed final step of branched chain amino acid biosynthesis supports intimate coordination between the aphid and *Buchnera* in completion of amino acid biosynthesis pathways (Hansen and Moran 2011), but also opens the metabolic pathway to host control. Recent work identifying microRNA (miRNA) targets in aphid bacteriocytes suggests that expression of BCAT is miRNA regulated, thereby implicating host regulatory checks in the production of the branched-chain amino acids (Feng *et al.* 2018). With respect to symbiont control, it is of interest that the genes for leucine biosynthesis are located on a plasmid within *Buchnera* from *M. persicae* and *A. pisum*, a genomic location that potentially uncouples leucine production rates from *Buchnera* genome copy number, a feature of symbiont genome evolution that possibly reveals some level of endosymbiont control of leucine production (Baumann *et al.* 1999; Jiang *et al.* 2013; Shigenobu *et al.* 2000). The fourth amino acid that feeds inputs into mTOR via Sestrin is methionine. Methionine also appears to be collaboratively synthesized by aphids and *Buchnera*, although in this case, the final biosynthesis step is predicted to be carried out in *Buchnera*, with intermediate metabolites provisioned by the aphid host (Russell *et al.* 2013).

Here we identified a putative Sestrin gene in the genomes of *M. persicae* and *A. pisum* (Figure 2). This putative Sestrin has not yet been characterized as leucine responsive (similar to the mammalian Sestrins 1 & 2, which are capable of binding leucine) or non-responsive (similar to the mammalian Sestrin 3, which is incapable of binding leucine) (Lee *et al.* 2016; Saxton *et al.* 2016; Wolfson *et al.* 2016). The single Sestrin found in *Drosophila* has been shown to be weakly responsive to leucine treatments (Wolfson *et al.* 2016), and it has been shown that genes downstream of the *A. pisum* mTORC1 are activated in response to leucine and methionine treatments (Gao *et al.* 2018). An amino acid responsive Sestrin (as proposed in Figure 1), a function conserved from *Drosophila* and present in mammals, would present a point of integration for these four collaboratively synthesized amino acids into mTOR signaling.

### Buchnera produced amino acids may be integrated into mTORC1 signaling by the SLC38A9/vATPase complex

One of the several points of signaling into mTORC1 occurs through the lysosomal vATPase/SLC38A9 complex that detects glutamine, arginine, and asparagine (Jewell *et al.* 2015; Wang *et al.* 2015; Yao *et al.* 2017). We found transcripts of both vATPase and SLC38A9 present and highly expressed in aphid bacteriocytes, and highly expressed in bacteriocytes relative to other aphid tissues (Figures 1, 3B, 4B, & 5). The non-essential amino acid glutamine plays an important role in aphid/*Buchnera* endosymbiosis as it is hypothesized to be used as a common precursor for *Buchnera*-produced amino acids (Sasaki and Ishikawa 1995). *Buchnera* from *M. persicae* (*Buchnera* Mp), unlike *Buchnera* from *A. pisum*, have retained a gene encoding an asparaginase in their genome, suggesting that *Buchnera* Mp might also be able to use host provisioned asparagine as an amino acid precursor in addition to glutamine (Jiang *et al.* 2013; Shigenobu *et al.* 2000). Host glutamine provisioning has previously been proposed to be regulated via the amino acid transporter ApGLNT1, that localizes to the bacteriocyte plasma membrane (Price *et al.* 2014). ApGLNT1 has high affinity for glutamine transport, but transport of glutamine is competitively inhibited by arginine (Price *et al.* 2014). Within the aphid/*Buchnera* endosymbiosis, arginine is produced by *Buchnera* (Hansen and Moran 2011; International Aphid Genomics Consortium 2010; Shigenobu *et al.* 2000). Host sensing of arginine via a SLC38A9 ortholog would integrate *Buchnera*’s output of host-essential amino acids into mTORC1 signaling, while SLC38A9 sensing of glutamine and asparagine would represent additional host controls on the aphid provisioning of universal amino acid precursors to *Buchnera*. Remarkably, SLC38A9 itself may be under another level of host control as it too is proposed to be a target of miRNA mediated regulation of gene expression in aphids (Feng *et al.* 2018). While aphid orthologs of SLC38A9 and vATPase are yet to be functionally characterized, or localized within bacteriomes, immunolocalization and functional characterization of these genes will reveal whether they colocalize and maintain their ancestral function in aphids.

### The mTOR pathway presents an additional novel mechanism of host/endosymbiont integration in the face of genetic constraint

Endosymbiosis entails a cost to the host of maintaining a symbiont within its cells. This cost will always exist for a host that houses an endosymbiont and therefore yields a situation that generates conflict between host and endosymbiont. In nutritional endosymbiosis, the endosymbiont appears to offset the cost it imposes on the host by providing a nutritional benefit to its host, a benefit that is accrued by the host at a cost to the endosymbiont. Thus, there exists a difference between what is good for the symbiotic relationship, and what is good for either member of a symbiotic relationship in isolation. Endosymbiosis can effectively be viewed as an antagonistic relationship where both members impose a cost upon each other, but in the right context they can provide a benefit to their partner that outweighs the cost (Keeling and McCutcheon 2017). Organisms in persistent endosymbiosis must evolve systems of integration and mediation to maintain mutually beneficial conditions (Leigh 2010). As described in the previous two sections, recent work in aphids reveals that multiple layers of regulation are implicated in the maintenance of beneficial conditions. These layers include within pathway metabolic collaboration (Russell *et al.* 2013), a negative feedback loop that regulates *Buchnera* amino acid precursor supply (Price *et al.* 2014), differential expression of *Buchnera* small RNAs (Hansen and Degnan 2014), aphid miRNA regulation of gene expression (Feng *et al.* 2018), and now mTOR.

In summary, we propose that the mTOR pathway, and specifically the amino acid sensing mTORC1, presents a compelling and logical potential candidate for an additional, novel system integrating host and symbiont within the aphid/*Buchnera* system. We further speculate that if mTOR integrates the aphid/*Buchnera* nutritional symbiosis, it will also function in host/symbiont integration in other systems. Both cooption of conserved genetic machinery and within pathway host/symbiont metabolic collaboration have emerged as clear and convergent signatures of host endosymbiont coevolution (Wilson and Duncan 2015). We predict that the highly conserved and nutrient sensitive mTOR pathway has been coopted in many collaborative nutritional endosymbioses as one among many integration systems, but one with important implications for regulation of endosymbiont populations within host cells. The essential next steps will require testing the model of integration we propose here through functional characterization of mTOR genes and pathway function in aphids.
